# Supplementary material for: Resolving the full spectrum of human genome variation using Linked-Reads
Source: Genome Res. 2019 Apr;29(4):635–45. doi: 10.1101/gr.234443.118 (PMC6442396; doi:10.1101/gr.234443.118)
Supplement: Supplemental Material [file supp_gr.234443.118_Supplemental_longranger-2.2.zip › longranger-2.2.tar.bz2!/lr2.2-public/lib/stan/cmdstan/stan/doc/api/html/structstan_1_1gm_1_1expression-members.html]

Stan: Member List


|  |  |
| --- | --- |
|  | Stan  2.5.0  probability, sampling & optimization |


- Main Page
- Related Pages
- Namespaces
- Classes
- Files

- Class List
- Class Index
- Class Hierarchy
- Class Members

All Classes Namespaces Files Functions Variables Typedefs Enumerations Enumerator Friends Macros Pages

- stan
- gm
- expression

stan::gm::expression Member List

This is the complete list of members for stan::gm::expression, including all inherited members.

|  |  |  |
| --- | --- | --- |
| expr\_ | stan::gm::expression |  |
| expression() | stan::gm::expression |  |
| expression(const expression &e) | stan::gm::expression |  |
| expression(const nil &expr) | stan::gm::expression |  |
| expression(const int\_literal &expr) | stan::gm::expression |  |
| expression(const double\_literal &expr) | stan::gm::expression |  |
| expression(const array\_literal &expr) | stan::gm::expression |  |
| expression(const variable &expr) | stan::gm::expression |  |
| expression(const fun &expr) | stan::gm::expression |  |
| expression(const integrate\_ode &expr) | stan::gm::expression |  |
| expression(const index\_op &expr) | stan::gm::expression |  |
| expression(const binary\_op &expr) | stan::gm::expression |  |
| expression(const unary\_op &expr) | stan::gm::expression |  |
| expression(const expression\_t &expr\_) | stan::gm::expression |  |
| expression\_t typedef | stan::gm::expression |  |
| expression\_type() const | stan::gm::expression |  |
| operator\*=(const expression &rhs) | stan::gm::expression |  |
| operator+=(const expression &rhs) | stan::gm::expression |  |
| operator-=(const expression &rhs) | stan::gm::expression |  |
| operator/=(const expression &rhs) | stan::gm::expression |  |


---

[ Stan Home Page ]

*© 2011–2014,
Stan Development Team.*
